# The Dutch multidisciplinary guideline osteoporosis and fracture prevention, taking a local guideline to the international arena

**DOI:** 10.1007/s11657-024-01378-3

**Published:** 2024-04-02

**Authors:** J. P. van den Bergh, P. Geusens, N. M. Appelman-Dijkstra, H. J. G. van den Broek, P. J. M. Elders, G. de Klerk, M. van Oostwaard, H. C. Willems, M. C. Zillikens, W. F. Lems

**Affiliations:** 1 Department of Internal Medicine, VieCuri Medical Center, Venlo, the Netherlands; 2 Department of Internal Medicine, Maastricht University Medical Center, Maastricht, the Netherlands; 3 Department of Internal Medicine, Subdivision Rheumatology, Maastricht University Medical Center, Maastricht, the Netherlands; 4 Department of Medicine and Life Science, Hasselt University, Hasselt, Belgium; 5 Department of Internal Medicine, Division Endocrinology, Leiden University Medical Center, Leiden, the Netherlands; 6 Osteoporose Vereniging, Bilthoven, the Netherlands; 7 Department of General Practice, Amsterdam Public Health Institute, Amsterdam UMC, Amsterdam, the Netherlands; 8 Department of Surgery, ADRZ, Goes, the Netherlands; 9 Department of Internal Medicine and Geriatrics, Amsterdam University Medical Center, Amsterdam, the Netherlands; 10 Department of Internal Medicine, Erasmus MC, University Medical Center, Rotterdam, the Netherlands; 11 Department of Rheumatology, Amsterdam University Medical Center, Amsterdam, the Netherlands

**Keywords:** Fracture prevention, Multidisciplinary guideline, Osteoporosis

## Abstract

**Background:**

In 2018, a grant was provided for an evidence-based guideline on osteoporosis and fracture prevention based on 10 clinically relevant questions.

**Methods:**

A multidisciplinary working group was formed with delegates from Dutch scientific and professional societies, including representatives from the patient’s organization and the Dutch Institute for Medical Knowledge. The purpose was to obtain a broad consensus among all participating societies to facilitate the implementation of the updated guideline.

**Results:**

Novel recommendations in our guideline are as follows:

- In patients with an indication for DXA of the lumbar spine and hips, there is also an indication for VFA.

- Directly starting with anabolic drugs (teriparatide or romosozumab) in patients with a very high fracture risk;

- Directly starting with zoledronic acid in patients 75 years and over with a hip fracture (independent of DXA);

- Directly starting with parenteral drugs (denosumab, teriparatide, zoledronic acid) in glucocorticoid-induced osteoporosis with very high fracture risk;

- A lifelong fracture risk management, including lifestyle, is indicated from the start of the first treatment.

**Conclusion:**

In our new multidisciplinary guideline osteoporosis and fracture prevention, we developed 5 “relatively new statements” that are all a crucial step forward in the optimization of diagnosis and treatment for fracture prevention. We also developed 5 flowcharts, and we suppose that this may be helpful for individual doctors and their patients in daily practice and may facilitate implementation.

## Introduction

In 2018, a grant from the Dutch Foundation Quality Funding of Medical Specialists (Stichting Kwaliteitsgelden Medisch Specialisten (SKMS)) was obtained at the request of endocrinologists to provide an update of the evidence-based guideline for osteoporosis and fracture prevention that was published in 2011, based on 10 clinically relevant questions. The grant proposal and the update were coordinated by the Dutch Society for Internal Medicine. After getting approval for financial support, a multidisciplinary working group was formed with delegates from Dutch scientific and professional societies to participate in the multidisciplinary working group. The core working group consisted of 3 internists, 3 general practitioners, 2 rheumatologists, a geriatrician, a trauma surgeon, a nurse practitioner, a member of the Dutch Osteoporosis Patient Association, and a senior advisor of the Quality Institute of the Federation of Dutch Medical Specialists. The core working group was responsible for the scientific update of the guideline. In addition, an advisory board was formed by delegates of various scientific societies (Pharmacy, Nurse Specialists, Physical Therapy, Dietetics, Sports Medicine, Gynecology, Radiology, Nuclear Medicine, Rehabilitation Medicine, Dental Surgery) to provide comments, reflections, and adaptations on all chapters of the updated proposal. The purpose was to obtain a broad consensus among all participating societies to facilitate the implementation of the updated guideline by all disciplines. The core working group started in 2019, and the updated version of the guideline was ready in July 2021 and was sent to all associations involved for review. The review period was open for 6 months. Based on the comments, the guideline text was extensively revised, and the revised version of the guideline was sent to all societies for final approval, which was obtained in August 2022. The guideline was authorized by the Federation of Dutch Medical Specialists in August 2022 and became available on the online Dutch Guideline database in September 2022: (https://richtlijnendatabase.nl/richtlijn/osteoporose_en_fractuurpreventie/startpagina_-_osteoporose_en_fractuurpreventie.html).

## Methodology

The development of this guideline was based on the AGREE (Appraisal of Guidelines for Research & Evaluation) II instrument (Brouwers, 2010). Briefly, for all 10 research questions, the PICO (Patient/Problem, Intervention, Comparison, and Outcome) strategy was utilized to search relevant literature in the Cochrane Library, PubMed, and Embase. To grade the quality of evidence and the strength of the guideline recommendations, the GRADE (Grading of Recommendations Assessment, Development, and Evaluation) approach was used. The evidence-to-decision framework from the GRADE Working Group was applied to translate the available evidence into recommendations [1, 2]. In Table 1, we showed the items that are relevant to the quality of the guideline/set of recommendations.

**Table 1:** Quality items of the Dutch multidisciplinary guideline osteoporosis and fracture prevention

Working group consists of official delegates of professional and scientific organizations, including patients organization
Multidisciplinary working group, with delegates from endocrinology, geriatrics, rheumatology, farmacology, general practitioners, nurse practitioners and patients
An advisory board was formed by delegates of various scientific societies (Pharmacy, Nurse Specialists, Physical Therapy, Dietetics, Sports Medicine, Gynecology, Radiology, Nuclear Medicine, Rehabilitation Medicine, Dental Surgery) to provide comments, reflections and adaptations
10 research questions, for which a literature search was done by a senior advisor of the Quality Institute of the Federation of Dutch Medical Specialists
To grade the quality of evidence and the strength of the guideline recommendations, the GRADE (Grading of Recommendations Assessment, Development and Evaluation) approach was used
We made 5 flowcharts showing what to do in which patient for diagnosis and treatment of osteoporosis, for educational reasons and to facilitate implementation

## Results

The literature search and the discussion led to several recommendations; 5 are, in our opinion, relatively “new in guidelines”, and of course, some are more or less in line with other guidelines. We report here on those 5 recommendations that are really “new in guidelines” (Table 2).

**Table 2:** What is really new in the Dutch Multidisciplinary Guideline Osteoporosis and Fracture Prevention

Diagnosis: In all patients with an indication for DXA of lumbar spine and hip, lateral imaging of the spine has to be performed with vertebral fracture assessment (VFA)
Treatment: Directly starting with anabolic drugs (teriparatide or romosozumab) in patients with a very high fracture risk
Treatment: Directly starting with second-line drugs (denosumab, teriparatide, zoledronic acid) in glucocorticoid-induced osteoporosis at very high fracture risk
Treatment: Directly starting with zoledronic acid in patients 75 years and over with a hip fracture (independent of DXA)
Management: A lifelong fracture prevention strategy is indicated from the start of the first treatment

### In patients with an indication for DXA of lumbar spine and hips, there is also an indication for VFA

Although fracture risk can be estimated by clinical risk factors and by DXA-BMD, with DXA-VFA, it is possible to diagnose vertebral fractures (VFs) [3]. With VFA, a quick visual assessment of the heights of the vertebrae of the lateral spine is performed in the same session as the DXA-BMD. DXA-VFA is a relatively cheap and reliable technique to detect vertebral fractures, particularly moderate to severe fractures (Genant Grades II and III).

Vertebral fractures (VFs) are the most common fractures among men and women in individuals 50 years and above [4–7]. It has been estimated that only 1 out of 3 or 1 out of 4 vertebral fractures is symptomatic [7, 8]. Thus, the majority of VFs are asymptomatic, defined as a vertebral fracture on a radiograph in an individual not seeking medical help from a physician because of back pain around the location of the vertebral fracture. These so-called asymptomatic vertebral fractures represent one of the important gaps in our ability to find all patients with high fracture risk, but they can be identified easily and reliably with DXA-VFA [9].

The clinical relevance of diagnosing vertebral fractures is that vertebral fractures are risk factors for future fractures, independent of BMD. In patients with VFs, the risk for both vertebral and nonvertebral fractures, (including hip fractures) is elevated [10, 11]. The risk of both incident, vertebral and non-vertebral fractures, is strongly related to the combination of low BMD and the number and severity of prevalent vertebral fractures [10, 11].

Thus, there are three reasons for performing a DXA-VFA in addition to DXA-BMD:

1. to diagnose one or more vertebral fractures, which elevates the future fracture risk;
2. to have a baseline value; when an osteoporotic patient suffers from back pain during treatment, it makes it possible to differentiate between a prevalent fracture (already present before starting drug treatment, thus no treatment failure) and a new vertebral fracture, which opens the discussion about a treatment failure;
3. in some patients in which moderate or severe vertebral fractures (Genant Grade 2 or more) can be diagnosed, this may result in an indication for anabolic therapy

Another step forward is the reporting of the results of VFA. In daily practice, a fracture on VFA is often described as a “little” or “substantial” fracture, which are vague and non-scientific terms. We invited the Dutch Societies of Radiology and Nuclear Medicine, in the Netherlands usually responsible for reporting for DXA and VFA, and discussed around a scoring method that is one of the best and easiest and reliable to implement nationwide: both the above-mentioned scientific societies and multidisciplinary guideline committee preferred that all vertebral fractures should be graded morphometrically according to the Genant method [12]. The grading according to the Genant is dependent on height loss: mild (Grade 1, 20 to 24%), moderate (Grade 2, 25 to 39%), and severe (40% or more).

### Anabolic drugs in high-risk postmenopausal women

Until recently, teriparatide was the only drug available in the Netherlands and was prescribed to men and women with at least two prevalent fractures who had another vertebral or nonvertebral fracture after at least 1 year of treatment with an antiresorptive drug. This has changed rigorously with the introduction of romosozumab and by two studies showing the superiority in fracture reduction of teriparatide over risedronate and romosozumab over alendronate [13, 14]. In the 2-year VERO trial, vertebral fractures occurred in 5.4% of teriparatide users, and in 12.0% of risedronate users (hazard ratio 0.44, 95% c.i. 0.29–0.68, *p* < 0.0001), and there was a trend for reduction in nonvertebral fractures: 4.0% versus 6.1% (hazard ratio 0.66, 95% c.i. 0.39–1.10) [14].

In the ARCH trial, the primary endpoints were the cumulative incidence of new vertebral fractures and clinical fractures (nonvertebral and symptomatic vertebral fracture) at 24 months. Vertebral fractures occurred in 6.2% of romosozumab users and 11.9% of alendronate users (hazard ratio 0.52, 95% c.i. 0.40–0.66, *p* < 0.0001, and the cumulative incidence of clinical fractures was also significantly lower in romosozumab users with 9.7% versus 13.0% (hazard ratio 0.73, 95% c.i. 0.61–0.88), including the risk of nonvertebral fractures with 8.7% versus 10.6% (hazard ratio 0.81 (95% c.i.:0.66–0.99) [13].

Thus, a new indication for anabolic treatment is based on the evidence that they have fracture prevention superiority as initial therapy above anti-resorptive drugs. This is in line with an earlier algorithm, in which it is advocated to use anabolic drugs as initial therapy in high-risk patients and that treatment should be continued with an antiresorptive agent after completion of the anabolic treatment period of 1 year with romosozumab and 2 years of teriparatide [15].

This is changing the paradigm, not waiting for fractures as a treatment failure, but starting with anabolics in those with the highest fracture risk. The reasons for only starting with anabolics in high-risk patients are the balance between the strong effect on fracture reduction versus the higher drug costs and the use of parenteral drugs instead of oral pills. However, although the concept of starting with anabolics is attractive, the definition of high fracture risk is arbitrary [15]. We choose for a high-risk definition based on the presence of low BMD combined with the presence of vertebral fractures as in the selection criteria of the VERO trial for teriparatide and the ARCH trial for romosozumab [13, 14].

Therefore, teriparatide should be considered first-choice treatment in postmenopausal women with a *T*-score ≤  − 1.5 in the FN, TH, or lumbar spine and at least 2 Grade 2 VFs or 1 Grade 3 VF.

Romosozumab should be considered first-line treatment in postmenopausal women without a history of MI or stroke and with *T*-score ≤  − 2.5 in the FN or TH (not lumbar spine) and at least 1 Grade 2 or 3 VF, or a *T*-score ≤  − 2.0 in the FN or TH (not lumbar spine) and at least 2 Grade 2 or 3 VFs. The guideline recommends that this level of care constitutes hospital-based care and underscored the importance of considering a hospital referral for patients meeting these criteria.

In such patients, anabolic treatment is recommended as the first choice; obviously, this is an enormous step forward in fracture prevention in high-risk patients with a low BMD and vertebral fractures.

### Directly starting with zoledronic acid in patients 75 years and over with a hip fracture (independently of DXA)

Hip fractures are associated with seriously reduced quality of life (the majority recover in a rehabilitation center) [16] and with high mortality risk: between 2016 and 2019, it was 28% in England and Wales [17]. Remarkably, many hip fracture patients are not treated with anti-osteoporotic drugs: when admitted to the hospital, there is an unnecessary fear of inhibiting fracture repair by using antiresorptives, and outside the hospital, no treatment is prescribed because patients are lost in follow-up, or it is too difficult to perform a DXA, etc. As a consequence, those with the highest fracture risk are not protected by antiosteoporotic drugs. Data from the UK showed that most patients (88.3%) are not taking any antiosteoporotic medication when they present with a hip fracture, and only half of patients (50.8%) were prescribed antiosteoporotic medication AOM treatment by the time of discharge, and the proportion deemed “inappropriate for AOM” varied hugely (0.2–83.6%) in different hospitals [18]. Another point is adherence to treatment, with maybe low in patients with osteoporosis, and particularly in the elderly, with polypharmacy. As multiple studies failed to provide evidence for altered negative fracture healing in these patients, we incorporated this option in the guideline. As a main body of evidence for this advice, we rely on the unique study from Lyles and colleagues, in hip fracture patients, in which zoledronic acid was given versus placebo within 90 days of the hip fracture: reduction in clinical vertebral fractures (− 46%), non-vertebral fractures (− 27%), and mortality risk (− 28%) was found [19]. Although this was only one (unique) study, the data are very convincing, and a strong argument to initiate zoledronic acid after a hip fracture in patients 75 years and over, even without a DXA/VFA. Of course, a DXA/VFA is relevant and can be performed later after the rehabilitation as a baseline value but is not necessary for decision-making about starting with antiosteoporotic treatment.

In individuals with a hip fracture below 75 years of age, we suggest the normal work-up as in other patients at the Fracture Liaison Service, with DXA/VFA, fall risk analysis, and screening for underlying osteoporosis.

### Directly starting with parenteral drugs (denosumab, teriparatide, zoledronic acid) in glucocorticoid-induced osteoporosis with very high fracture risk

Glucocorticoid-induced osteoporosis (GIOP) is the most common cause of secondary osteoporosis, and fracture risk in patients treated with glucocorticoids (GC) is related to primary risk factors for osteoporosis (age, low BMI, familiar osteoporosis, smoking, etc.) and risk factors for secondary osteoporosis (dosage of GC and severity of underlying disease) [20]). However, GC-treated patients are often undertreated; only 30–50% of GC-treated patients treated with prednisone 7.5 mg per day or more receive anti-osteoporotic drugs [21, 22]. We think that we can improve fracture reduction in GC-treated patients with the implementation of two strategies:

In patients 50 years and over, treated with 7.5 mg prednisone per day or more, we recommend direct starting with oral bisphosphonates, without waiting for the results of DXA/VFA. Nevertheless, it is important to perform a DXA/VFA even after starting with oral bisphosphonates, because when patients suffer from back pain 2 years later, the only way to differentiate between an old, already at baseline existing prevalent vertebral and a new, incident vertebral fracture, is to compare baseline with follow-up VFA.

A completely new issue is the starting with parenteral drugs in GC-treated patients at very high fracture risk, more or less analogous to the start with bone-forming agents (romosozumab, teriparatide) in postmenopausal women with very high fracture risk. Remarkably, in earlier studies, teriparatide has been proven to be superior to alendronate (favorable response in BMD and in vertebral fracture reduction [23], while both zoledronic acid [24] and Dmab [25] have been shown superior in changes in BMD versus risedronate. Thus, in our opinion, the start with oral bisphosphonates in GC-treated patients with a very high fracture risk is suboptimal, since more powerful drugs are available. Since there are no comparative trials between zoledronic acid, dmab, and teriparatide in GC-treated patients, we have no preference between these drugs and suggest physicians to choose between these 3 drugs based on the preferences and characteristics of the individual, very high-risk patient.

**Fig. 1:**
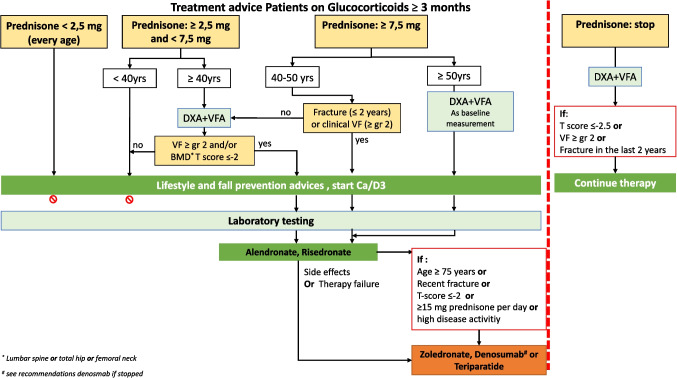
Treatment advice for patients treated with glucocorticoids

In the multidisciplinary guideline, very high risk in GC-treated patients (arbitrarily) defined as age > 70 years, and/or recent non-vertebral fracture (less than 2 years), and/or a vertebral fracture Grade 2 or more (> 25% height loss), and/or *T*-score < 2.0 in spine and/or hips, and or prednisone dosage > 15 mg for at least 3 months, and/or in patients with very severe disease activity (Fig. 1).

To stimulate the adequate use of anti-osteoporotic drugs, we made a scheme for clinicians that might be very helpful for GC-treated patients at various risks for fractures, with the age of the patient and the dosage of GC are the key elements of fracture risk. Apart from direct starting with bisphosphonates in high-risk patients and with second-line drugs in patients with very high risk, we also added a stopping rule, which is also relatively new: it is recommended that after tapering and stopping GC, DXA/VFA should be done, and treatment should be continued in patients with a *T*-score <  − 2.5, and/or a vertebral fracture grade 2, and/or a recent nonvertebral fracture (< 2 years).

### A lifelong fracture prevention strategy is indicated from the start of the first treatment

In many studies, it has been shown that adherence to treatment is one of the biggest issues in the field of osteoporosis, e.g., in the Netherlands, it has been shown that 1 year after starting with several oral anti-osteoporotic drugs, 50% had stopped [26]. Other data showed that for parenteral drugs, such as zoledronic acid, teriparatide and denosumab, adherence is also an issue: 2 years after starting therapy, around 50% has stopped [27]. Obviously, this is insufficient in the prevention of fractures. Analogous to cardiovascular risk management, which is lifelong, we propose a lifelong fracture risk management strategy in patients with high fracture risk. It is crucial to communicate to patients with a high fracture risk that they should have not 3 to 5 years of drug treatment, but a lifelong fracture management, which does not mean that they should always need medications since, e.g., bisphosphonates, there can be a drug-holiday after some years of treatment. It is important to realize that lifestyle measures, such as adequate nutrition, including calcium, vitamin D, and exercise/fall prevention play a crucial and complementary role, in addition to drug therapy.

**Fig. 2:**
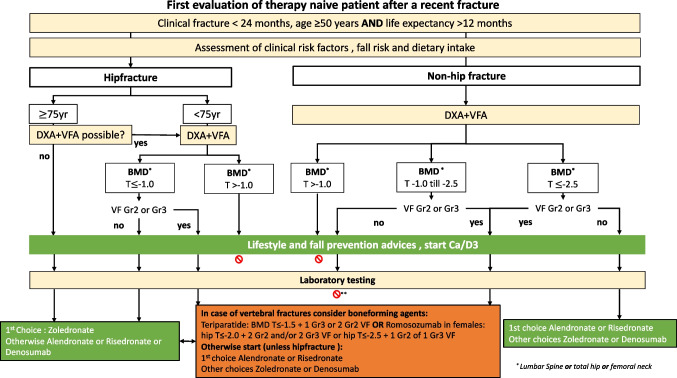
Evaluation of therapy-naive patient ≥ 50 years with a recent fracture

**Fig. 3:**
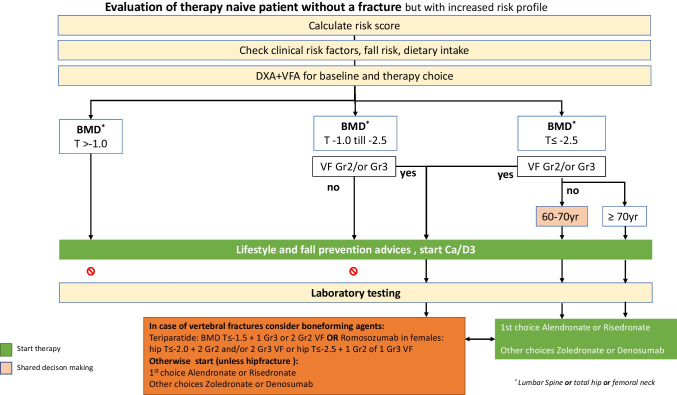
Evaluation of therapy-naive patient without a recent fracture, but with an increased risk profile

After the 4-step diagnostic process, oral weekly alendronate and risedronate are usually the first choices [28–30], while in case of GI complaints, alendronate in fluid form or zoledronate or denosumab is the best alternative and eventual raloxifene. As mentioned above, anabolic treatment (with teriparatide or romosozumab) is recommended as the first choice in patients with a combination of low BMD and (severe) VFs, and zoledronic acid is the first choice after a recent hip fracture in patients 75 years and over, even without a DXA and VFA. We have shown this in a flowchart in patients ≥ 50 years with a recent fracture (Fig. 2) and in a flowchart in patients without a recent fracture and without GC use, but with an increased risk profile based on BMD and prevalent VFs (Fig. 3). In this group of patients, the recommendations to start treatment are marked in green. In the subgroup of patients aged 60–70 years, it is not recommended to start treatment in all patients. In these patients, without a recent fracture or prevalent VFs and not using GC, the absolute fracture risk and, therefore, risk reduction with treatment may be low, and the decision to initiate treatment is mainly based on shared decision-making.

**Fig. 4:**
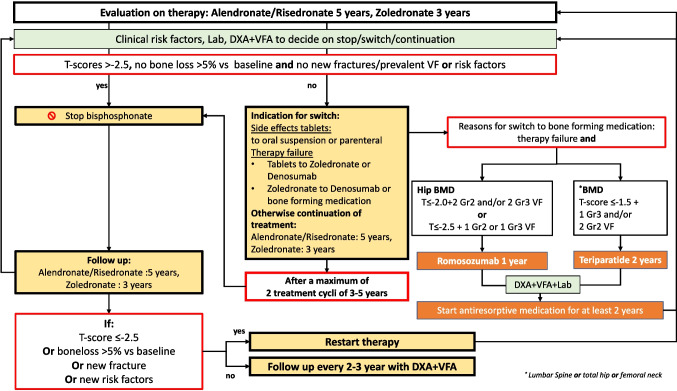
Evaluation on therapy: bisphosphonates

In a model of lifelong fracture risk management, monitoring is crucial. During any drug treatment, evaluation after 3 months and yearly afterward is recommended to discuss tolerance, compliance, and motivation and to eventually optimize compliance. Standard therapy in terms of duration is based on the results of fracture efficacy in pivotal RCTs and is recommended after 5 years for alendronate and risedronate [31, 32], after 3 years for zoledronate [33] (Fig. 4) and denosumab, after 2 years for teriparatide and after 1 year for romosozumab.

**Fig. 5:**
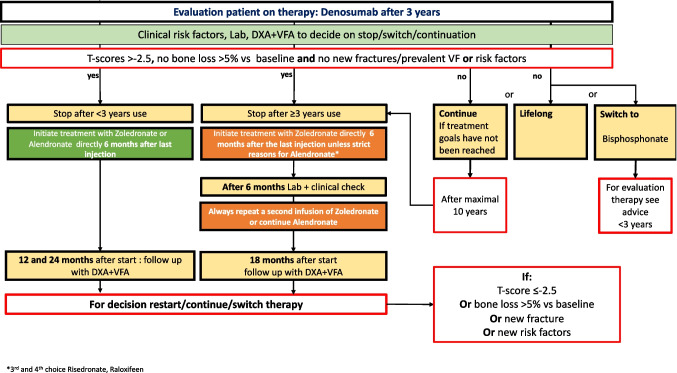
Evaluation on therapy: denosumab

It is extremely important to realize that while a 2–3 year drug holiday is possible in adequately treated bisphosphonate-treated patients, with other drugs, such as romosozumab and teripartide, the gain in BMD should be preserved by continuing with antiresorptive drugs. This is even more crucial for patients using denosumab: after stopping denosumab, the strongly depressed CTX-levels during denosumab do not gradually increase over the years to baseline but increase within a few months to above the baseline value (an overshoot reaction) and that is associated with an increase in vertebral fractures, particularly in those with prevalent fractures. Thus, for denosumab users, it is crucial to start with another drug not longer than 6 months after the last denosumab injection; it has been suggested that zoledronic acid might be the most effective, one injection, or a second injection 6 months later in patients with high fracture risk after 3 years or more denosumab use. See the flowcharts on evaluation of treatment with bisphosphonates (Fig. 4) and of denosumab (Fig. 5). For denosumab, a maximum of 10 years of continued treatment is recommended, as there is no evidence in the literature of safety and efficacy beyond this period of treatment.

Finally, reevaluation after drug therapy consists of a standard evaluation to decide about the level of future fracture risk. Low risk is defined as a *T*-score > − 2.5 (lowest T-score at the lumbar spine (LS) + femoral neck (FN) + total hip (TH), and no incident VF or NVF and no new clinical risk factors. Increased risk is defined as a *T*-score ≤  − 2.5 (lowest T-score at the LS or FN of TH), or bone loss > 5% (spine or FN of TH) or incident new VF or NVF, or new clinical risk factors. When the fracture risk is low, bisphosphonates and raloxifene should be stopped, and lifestyle and adequate calcium and vitamin D intake should be continued. Standard reevaluation is recommended 2 years after stopping oral bisphosphonates and raloxifene and 3 years after stopping zoledronate.

### Summary of the other clinically relevant questions and literature searches

As mentioned, the new Dutch guideline was based on a literature search of 10 clinically relevant questions and literature searches. For completeness, we report on all of them, but in a summarized form, because they are more or less in line with other guidelines and recommendations, and not so “new” as the above presented 5 topics.

Identification of patients with a high fracture risk is indicated in 3 groups

- 1. All patients 50 years and over with a recent fracture should be considered for evaluation at the Fracture Liaison Service (FLS). All these patients should be identified and invited for DXA/VFA, fall risk analysis and testing for forms of secondary osteoporosis. This is in line with current recommendations and guidelines from ASBMR, IOF, and EULAR [34–36], and probably also with other local guidelines. The biggest issue is the implementation: in 2016, a nationwide survey was done in the Netherlands, and only in 26% of fracture patients 50 years and over, a DXA was performed. Lack of awareness of osteoporosis among both patients and health professionals as a serious disease was one of the most important reasons, apart from financial issues and lack of clarity on the tasks of different health professionals in fracture prevention. Clearly, we must do better, one of the options is to start with fracture-prevention teams (with a surgeon, a non-surgeon (endocrinologist, rheumatologist, etc., and a fracture nurse) in all hospitals, who identify all fracture patients 50 years and over. Currently, nearly all of the around 80 hospitals in the Netherlands have an FLS, but undercapacity, particularly in fracture nurses, plays an important role in many hospitals.
- 2. In patients 60 years and over, with clinical risk factors, but without a recent fracture, and without the use of glucocorticoids. Based on our literature search, we made a table of risk factors, in patients with 4 or more points, a fracture risk analysis is indicated (DXA/VFA, fall risk analysis and testing for secondary osteoporosis).
  Although our risk factor table does not fully overlap with other scoring systems, such as FRAX, there is remarkable overlap. The selection of these clinical risks was based on the Dutch fracture risk data [37] (Table 3).
- 3. Patients treated with glucocorticoids, as discussed above.
- 4. Which laboratory testing should be performed in patients with an elevated fracture risk?

**Table 3:** Clinical risk factor scoring

Risk factors	Risk points
BMI < 20	1
Age > 60	1
Age > 70	2
Recent fracture	2
Fracture > 2 years ago	1
Parent with hip fracture	1
More than 1 fall event last year and/or immobility	1
Smoking and/or 3 or more alcoholic drinks per day	1
Comorbidities or drugs that interfere with bone metabolism	1

This screening includes serum calcium, albumin, creatinine, TSH, and 25(OH)vitamin D in all patients, with additionally upon clinical indication, e.g., severe osteoporosis/multiple fractures/fractures at a young age, ESR, M-protein electrophoresis, serum phosphate, alkaline phosphatase, testosterone in male individuals ≤ 70 years of age. We advise to perform laboratory testing in all patients with an indication for treatment but also in patients with a fracture and osteopenia. This was incorporated due to the outcomes of 2 Dutch studies in FLS patients showing that the secondary causes of osteoporosis not only occur in patients with *T*-scores ≤  − 2.5 but also in patients with osteopenia [38, 39].

- 5. What is the optimal screening method for screening for fall risk in patients with high fracture risk?

Fall risk is strongly age-related and a fall event is a strong risk factor for future fractures. Recently, it was shown in a 3-year observational cohort study at the FLS that an incident fall was associated with an approximately ninefold (HR: 8.6, 95% c.i. 3.1 to 23.8) increase in the risk of subsequent fractures [40]. Therefore, in patient with a high fracture risk due to falls, as in elderly individuals 65 years and over and a recent fall or fall-related fracture, it is advocated to perform a multifactorial risk analysis, usually by referring a patient to a fall-risk outpatient clinic.

For other patients with a lower fracture risk, such as individuals < 65 years of age and with 2 or more falls in recent year, it is advocated that the physician or fracture nurse checks for risk factors that can, at least theoretically, be influenced: alcohol intake, use of sedatives and other drugs (cardiovascular) that may increase fall risk and orthostatic hypotension, deteriorated vision, muscle, and balance weakness.

Interestingly, the authors expect that implementation will be difficult because of barriers in health care professionals: they do not regard falls as an important and modifiable risk factor for fractures. Moreover, implementing tests during the outpatient clinic visit for orthostatic hypotension and muscle and balance weakness is perceived as taking too much time. It is suggested to educate healthcare professionals performing the fracture liaison service with short training sessions, but one must realize that implementing these fall prevention strategies takes some time, thus is costly.

Last but not least, the healthy lifestyle. The issue of a healthy lifestyle was brought up by the patients; they had a strong preference to incorporate these topics in the 10 research questions of the new guideline. As a consequence, we discussed three topics, on calcium and vitamin D (as in the previous guideline), and we did three new searches on supplements with K2 and magnesium and on the effects of exercise on bone.

- 6. How much calcium and vitamin D are necessary for patients with a high fracture risk?

For calcium, we recommend in all individuals with an elevated fracture risk, as before, an intake of 1000–1100 mg per day, and thus 500 mg or 1000 mg calcium supplementation in patients with only 2–3 or less than 2 calcium products (milk, yoghurt, cheese or calcium-containing vegan alternatives) per day, respectively.

For vitamin D, we recommend in all individuals with an elevated fracture risk, as before, the daily use of 800 IU vitamin D. However, we discourage the use of high daily dosages (3000 IU or more per day) of bolus regimes (> 60.000 IE per month) because these are probably related to an increased fall risk [41–43].

- 7. Are supplements of magnesium and/or vitamin K useful in patients with high fracture risk?

Magnesium probably plays a role in the mineralization of bone and is essential in many cellular processes. In some studies in elderly individuals, a low magnesium intake was associated with low BMD [44, 45], but not in other studies, and in a meta-analysis, only a weak association was found for magnesium and hip BMD, but not with lumbar spine BMD and with fractures [46]. Since no randomized studies have been performed on the effects of magnesium supplementation on bone, we recommend for individuals with an elevated fracture risk a healthy diet, with adequate calcium, vegetables fruit and nuts intake, but no magnesium supplementation.

Vitamin K has, apart from its effect on blood coagulation, also an effect on bone mineralization and bone formation. However, in a meta-analysis, no effect of vitamin K supplementation was found on BMD and on fracture rate [47]. It is important to realize that vitamin K supplementation might interfere with blood coagulation. In line with the advice for magnesium, we recommend healthy food and no vitamin K supplementation in patients with a high fracture risk.

- 8. Is exercise therapy useful in patients with high fracture risk?

Randomized controlled trials in osteopenic and/or osteoporotic patients observing the effects of exercise therapy on fall risk and fractures are rare, probably because large populations and long-term follow-up are necessary, which is difficult to organize and costly (without sponsoring from pharmaceutical companies). Nevertheless, in our opinion, exercise is crucial not only for the heart, lungs and muscles but also for healthy and strong bones.

For clinicians, it is recommended to evaluate and discuss the exercise behavior of the patient with an elevated fracture risk and to check whether it is in line with the Dutch exercise directive: it is advocated to perform at least 2.5 h per week (150 min) on different days, weight-bearing physical activities, such as walking, and twice a week exercises for muscle strengthening, such as balance and fall prevention exercises, and/or brisk walking. For some (frail) individual patients, a referral to the physical therapist can be useful.

We think that more attention to lifestyle is important in the optimization of therapy for patients with a high fracture risk, and thus, we support that 3 out of 10 items in the new multidisciplinary guideline focus on lifestyle. However, we also see patients who embrace the lifestyle recommendations but are still very worried about the side effects of anti-osteoporotic drugs. It is considered critical that the combination of a healthy lifestyle and the adequate use of antiosteoporotic drugs is the optimal strategy to reduce fracture risk. Preliminary recent studies show that the combination of exercise programs with antiresorptive drugs could be more efficient in raising BMD than either alone [48, 49].

- 9. Organization of care

Many professionals play a role in the care of patients with a high fracture risk: medical specialists (endocrinologists, rheumatologists, geriatricians, orthopedics/traumatologists), fracture nurses, general practitioners, and others. Although the type of professionals involved in the care of osteoporotic patients differs from country to country, we thought that we should make a model of care for osteoporotic patients in our country. In the previous Dutch guideline, from 2011, we had the opinion that everyone who is willing and capable to do the 4 steps diagnostic model, either medical specialist or general practitioner, should be allowed to do that. However, in daily practice, no one feels responsible for these fracture patients in some regions. Therefore, we developed a more strict model of care for fracture patients. The first steps, identifying patients 50 years and over with a recent fracture, followed by a 4-step diagnostic model and initiation of treatment in high-risk patients is the responsibility of the fracture-prevention team of the hospital. The fracture prevention team preferably consists of 3 professionals: an orthopedic surgeon/traumatologist, an endocrinologist/rheumatologist/geriatrician, and a fracture nurse. Nowadays, adequately functioning fracture prevention teams are not yet nationwide available, but it is at the top of the implementation agenda.

What to do after treatment initiation? A follow-up moment after 3 months is advocated, checking for adherence to therapy, possible side effects of the drugs, and the possibility for the patients to ask additional questions on the drugs, healthy lifestyle, and prognosis. After that, the patients are referred back to their general practitioner, for the rest of the 5-year period of drug treatment with alendronate or risedronate.

Drug treatment with zoledronic acid and denosumab is usually initially for 3 years; in some regions, patients will be referred back to their general practitioners; in other situations, they will remain under the specialized care of the fracture prevention team.

- 10. Implementation

No one should be surprised when a high-quality set of recommendations has only a very small impact on daily practice; thus, an implementation plan should be an integral part of the document. Implementation of recommendations and guidelines in daily practice are among the biggest issues in modern medicine; both facilitators and barriers play a role [50]. What are the facilitators and barriers in our new guideline? We suppose that the literature search leading to evidence-based recommendations, the multidisciplinarity of the working group, including not only medical specialists but also general practitioners, and representatives of fracture nurses and patients, and the 5 flowcharts, may facilitate the implementation of the guideline. However, there are also barriers that may play a role, among them a lack of awareness of the clinical consequences of osteoporosis and fear of side effects of drug treatment, etc. We made a table of several key issues that may limit the implementation, with corresponding possible solutions to improve implementation. The main key issues on our list were underdiagnosis in high fracture-risk patients, undertreatment, lack of awareness among the public and patients, and external barriers, including among them financial issues [51]. In our opinion, the first step of the implementation of a guideline or set of recommendations is to make an analysis of barriers and facilitators and, based on that, to start with an agenda to strengthen the facilitators and to weaken the role of the barriers.

## Discussion

Local recommendations and guidelines are made in many countries in their local language. Usually, there are some differences between countries, related to differences in health care system including access to DXA/VFA and to osteoporotic drugs, but also to differences in fracture risk, life expectancy, and sometimes also a “colour locale”. Nevertheless, there are also similarities, and the most important one is that all recommendations and guidelines strive to reduce fracture incidence.

In our opinion, it is useful to bring country-specific recommendations and guidelines in the international arena; it may improve communication between countries, and we can learn from each other. We suppose that the quality of the process of developing and approving of the guideline, the results with 5 statements that are (relatively) “new in guidelines’’, and the flowcharts are helpful to clinicians in daily practice.

We hope that others will do the same with their local recommendations and guidelines that will open up possibilities to compare the different recommendations and guidelines, and probably also existing differences may grow toward each other over the years when making new sets of recommendations/guidelines.
